# Trends in infant nutrition in Saudi Arabia: compliance with WHO recommendations

**DOI:** 10.4103/0256-4947.51812

**Published:** 2009

**Authors:** Mohammed I. El Mouzan, Ahmad A. Al Omar, Abdulla A. Al Salloum, Abdulla S. Al Herbish, Mansour M. Qurachi

**Affiliations:** a From the Department of Pediatrics, King Saud University, Riyadh, Saudi Arabia; b From the The Children's Hospital, Riyadh Medical Complex, Riyadh, Saudi Arabia; c From the Department of Pediatrics, Al Yamama Hospital, Riyadh, Saudi Arabia

## Abstract

**BACKGROUND AND OBJECTIVE:**

The WHO recommends exclusive breastfeeding in the first 6 months of life. Our objective was to evaluate trends in infant nutrition in Saudi Arabia and the degree of compliance with WHO recommendations.

**SUBJECTS AND METHODS:**

A nationwide nutritional survey of a sample of Saudi households was selected by the multistage probability sampling procedure. A validated questionnaire was administered to mothers of children less than 3 years of age.

**RESULTS:**

Of 5339 children in the sample, 4889 received breast milk at birth indicating a prevalence of initiation of 91.6%. Initiation of breastfeeding was delayed beyond 6 hours after birth in 28.1% of the infants. Bottle feeding was introduced by 1 month of age to 2174/4260 (51.4%) and to 3831/4260 (90%) by 6 months of age. The majority of infants 3870/4787 (80.8%) were introduced to “solid foods” between 4 to 6 months of age and whole milk feedings were given to 40% of children younger than 12 months of age.

**CONCLUSIONS:**

The current practice of feeding of Saudi infants is very far from compliance with even the most conservative WHO recommendations of exclusive breastfeeding for 4 to 6 months. The high prevalence of breastfeeding initiation at birth indicates the willingness of Saudi mothers to breastfeed. However, early introduction of complementary feedings reduced the period of exclusive breastfeeding. Research in infant nutrition should be a public health priority to improve the rate of breastfeeding and to minimize other inappropriate practices.

Numerous studies have demonstrated the advantages of breastfeeding infants. In fact, breastfeeding is considered to be beneficial for both infants and mothers and recommendations for breastfeeding have been issued by professional agencies and societies including the American Academy of Pediatrics, the United Nation Children Funds (UNICEF) and the American Dietetic Association.^1^–^3^ The World Health Organization (WHO) has recommended that infants be exclusively breastfed for 4 to 6 months with the introduction of complementary food thereafter.^4^ However, based on a WHO expert consultation regarding the optimal duration of exclusive breastfeeding,^5^ this recommendation was changed in 2001 to extend the period of exclusive breastfeeding for 6 months.^6^ The objective of this study was to evaluate the trend in the pattern of infant nutrition in Saudi Arabia and to demonstrate the extent of compliance with the WHO recommendations.

## SUBJECTS AND METHODS

The present nationwide nutritional survey was performed as part of the Health Profile for Saudi Children and Adolescents Projects. A detailed description of the methodology has been reported.^7^ Briefly, households were randomly selected using a stratified multistage probability sampling procedure from a listing based on the 1992 census (updated in 2001). This process was completely computerized. It was performed with the assistance of the General Directorate of Statistics, Ministry of Planning. The sample was cross-sectional and therefore no follow-up data was collected. The nutritional section of a validated questionnaire was administered to all mothers of healthy children less than 3 years of age at the time of the survey (2004-2005), after obtaining their consent for the interview. It included information on initiation and timing of breastfeeding after birth, the reasons for not breastfeeding for those who were not breastfed or stopped breastfeeding, age at introduction of bottle milk formula feeding, feeding whole milk (cow, goat or camel) and the introduction of solid food. A trained member of the field team administered the questionnaire to mothers by direct interview during house visits. Simple descriptive statistics are used to present the results.

## RESULTS

From 2004 to 2005, the mothers of 5339 children who were less than 3 years of age were interviewed. There was a history of breastfeeding in 4889 (91.6%) and 450 (8.4%) were formula fed and therefore never breastfed (Table 1). Only 23.2% of newborns were initiated on breastfeeding within the first hour after birth, and breastfeeding was delayed beyond 6 hours after birth in 28.1%. The prevalence of combined breast and milk formula feeding (Table 2) indicates a drop from 88.6% at birth to 1.8% at 12 months. Of the 772 mothers who had stopped breastfeeding at the time of the survey, the commonest cause given was insufficient milk in 351 (45.5%), followed by illness of the mother, breast problems and illness of the baby (Table 1). Nearly 80% and 90% of the infants were started on bottle milk formula by 4 and 6 months, respectively (Table 3). “Solid” food other than milk was introduced to 81.5% of infants between 4 to 6 months of age. Finally, about 40% of the infants younger than 12 months of age were fed whole milk.

**Table 1:** Pattern of breastfeeding in nationwide nutritional survey of 5339 children less than 3 years of age.

	Number (%)
Initial feeding	
Breastfed	4889 (91.6)
Bottle fed (never breastfed)	450 (8.4)

Timing after birth (hours)	
<1	1134 (23.2)
1-3	1334 (27.3)
4-6	1048 (21.4)
>6	1373 (28.1)

Age prevalence of breastfeeding (months)	
Birth	3781 (88.6)
1	2092 (49.0)
2	1541 (36.1)
4	876 (20.5)
6	435 (10.2)
12	76 (1.8)

Reasons for stopping breastfeeding	
Mother had insufficient milk	351 (45.5)
Mother was ill	171 (22.1)
Breast problems/going to work	92 (11.9)
Baby was ill	64 (8.3)
others	94 (12.2)

**Table 2:** Prevalence of combined feedings in a nationwide nutritional survey.

Age (m)	Number bottle fed	Number breastfed	Prevalence (%)^a^
Birth	485	3781	88.6
1	2174	2092	49.0
2	2725	1541	36.1
4	3390	876	20.5
6	3831	435	10.2
12	4190	76	1.8
18	4251	15	0.4
24	4260	6	0.2

a Prevalence at any age=total no. of children–no. bottle-fed/total no. of children.

**Table 3:** Pattern of complementary feeding in a nationwide nutritional survey.

Introduction of complementary feeding (age in months)	Number (%)
Bottle feeding	
Birth	485 (11.4)
1	2174 (51.0)
2	2725 (63.9)
4	3390 (79.5)
6	3831 (89.8)
12	4190 (98.2)
**Total**	**4266 (100)**

“Solid food”	
<4	200 (4.2)
4-6	3870 (81.5)
7-12	680 (14.3)
**Total**	**4750 (100)**

Whole milk	
<6	78 (18.4)
6-11	91 (21.4)
12+	256 (60.2)
**Total**	**425 (100)**

## DISCUSSION

The recognition of the importance of early nutrition has led to increasing research on human milk. The results of this research indicate that human milk should be the standard and exclusive infant feeding in the first months of life. These facts led to the initial recommendation of the WHO that infants be exclusively breastfed for 4 to 6 months before introducing any other fluid or food, with continuation of breastfeeding up to 2 years. According to the WHO definition, exclusive breastfeeding means no other food or fluids (including plain water and juices). Infant milk formulas are considered complementary food.^8^ In 2001, the WHO extended the period of exclusive breastfeeding to 6 months instead of the period 4 to 6 months. However, there is still debate about the optimal duration of exclusive breastfeeding.^9^,^10^

Early studies on infant nutrition in Saudi Arabia have described some aspects of the pattern of infant feeding. In 1988, Al-Frayh et al reported on infant feeding practices in 4796 infants under 1 year of age living in Riyadh and found that the average duration of breastfeeding was 5.05 months, while bottle feeding was started under 1.0 month of age in 27.3% and the mean age for starting solid food was 3.5 months.^11^ In another nutritional survey conducted in several semirural areas of Saudi Arabia, Al-Othaimeen et al found that 21.5% of 767 children were breastfed completely, 68.4% used mixed feedings and about 10% did not breastfeed.^12^ In more recent reports, Al-Jassir et al reported data collectedfrom September 1999 to September 2000, from 21 507 infants and children less than 5 years attending primary health care centers in Riyadh and found that 98.9% were started on breastfeeding during the first week after birth and that breastfeeding was continued for more than 6 months in 52.7%. The mean duration of breastfeeding was 6.57 months, the mean age at introduction of bottle feeding was 1.84 months, and by the end of the first month 77.2% of the infants were bottle fed, and the mean age of weaning (solid food) was 4.43 months. The main reason for starting bottle feeding was “not enough milk” in 66.1%.^13^ The very high rate of initial breastfeeding in the latter study is similar to the 98% reported by Madani et al from Jeddah.^14^ In another survey of a random cross-sectional sample of mothers attending Al Kharj Health Center (Al-Kharj Military Hospital) from 1 November 2000 to 28 February 2001, Ogbeide et al reported that partial breastfeeding was the most common mode of infant feeding in 66.1%, and 27.3 % of mothers engaged in exclusive breastfeeding.^15^ This rate of exclusive breastfeeding is higher than previous reports, but the authors did not give the length of the period of exclusive breastfeeding making comparison difficult. In another survey carried out at King Abdulaziz Medical City in Riyadh (National Guard) primary care centers, Al-Hreashy et al reported a high rate of breastfeeding initiation (95%) and a high rate of milk formula supplementation and fluids in 83.4% and 94% during the first 6 months, respectively, and a very low rate (1.7%) of exclusive breastfeeding at 6 months.^16^

These studies are local and although they reflect prevalent patterns locally, they are not representative of infant feeding patterns in Saudi Arabia. Three previous community-based nationwide representative surveys have been reported, with the oldest survey conducted in 1987.^17^–^19^ The results reported by Al-Mazrou et al in 1994 indicated that at 1 month of age and younger, 55% were breastfed only, 40% were on breast and bottle, and 5% were on bottle only. By 6 months the percentages were 33%, 55% and 12%, respectively.^17^ However, the authors did not mention the rate of breastfeeding initiation at birth nor the exact definition of breastfeeding, only whether it was exclusive or not. The mean age at supplementation with solids was 5.3 months.

The next national survey was carried out in 1991 and reported by Al-Shehri et al in 1995. The prevalence of breastfeeding at the end of the first month was 93%, declining to 78% at the end of the sixth month and 45% at the end of first year. Breastfeeding only was present in 53% of infants younger than 5 months while breast and bottle were used among 34% and bottle only among 13% of the children. The authors did not mention the rate of initiation at birth and no definition of breast feeding only was provided. Milk formula was introduced to 71% in urban and 65% in rural areas, and solid food was introduced to 78% and 76% of the infants at the age 3-6 months in urban and rural areas, respectively. Compared to the previous survey, the results of this study indicated a higher breastfeeding rate at 1 month, and an earlier as well as a slower decline in the breastfeeding rate with age.^18^ The third national survey was the Saudi Arabia Family Health Survey conducted in 1995 and reported by Khoja and Farid in 2000. The main findings included an 87% rate of breastfeeding, with only 31% of infants younger than 4 months of age exclusively or predominantly breastfed. Bottle feedings were started in 33%, 10.1% and 8.4% of infants less than 3, 3 to 5 and 6 months of age and older, respectively. The commonest age of introduction of solid food was between 3 to 5 months in 51.6% of children, followed by 6 to 8 months of age in 23.7%.^19^

Our results are quite consistent not only with those of the nationally-representative surveys, but also with the other more recent local surveys. There is a very high prevalence of initiation to breastfeeding despite delay in initiation after delivery, indicating a willingness of Saudi mothers to breastfeed. Unfortunately, there is a clear tendency to introduce artificial milk formulas too early, leading to a parallel fast reduction and subsequent failure of breastfeeding. This high initiation rate is much higher than the US Healthy People 2010 goals of 75% initiation.^20^ However, our rates of exclusive and continued breastfeeding are lower than the rates of 42.5% for exclusive and 51.5% for mixed (breast and formula) feeding in US children at 3 months, and the rates of 13.3% for exclusive and 35.1 % for mixed breastfeeding at 6 months.^21^ In previous reports and in the present report, the rate of exclusive breastfeeding by age is not known, but judging from the age at introduction of bottle milk formula, this rate must be very low and far from both the old WHO recommendations of exclusive breastfeeding for 4 to 6 months and very far from the new recommendations calling for exclusive breastfeeding for 6 months.^22^,^23^

We conclude that the pattern of infant nutrition in Saudi Arabia has not changed much over the last two decades. Unless the true causes of the premature introduction of milk formula bottle feeding are identified and corrective measures undertaken, we will remain very far from compliance with even the old WHO recommendations. The Saudi Public Health Authorities and other research funding institutions such as King Abdulaziz City for Science and Technology should give priority to research in the important field of infant nutrition.
